# Correction to “LncRNA MYLK antisense RNA 1 activates cell division cycle 42/Neutal Wiskott–Aldrich syndrome protein pathway via microRNA‐101‐5p to accelerate epithelial‐to‐mesenchymal transition of colon cancer cells”

**DOI:** 10.1002/kjm2.12893

**Published:** 2024-09-04

Quan Z‐H, Xu F‐P, Huang Z, Chen R‐H, Xu Q‐W, Lin L. LncRNA MYLK antisense RNA 1 activates cell division cycle 42/Neutal Wiskott–Aldrich syndrome protein pathway via microRNA‐101‐5p to accelerate epithelial‐to‐mesenchymal transition of colon cancer cells. Kaohsiung J Med Sci. 2024;40(1):11–22. https://doi.org/10.1002/kjm2.12773.

The authors have found two errors in the manuscript that needs to be corrected:

1. On page 12, “First Affiliated Hospital of China Medical University” should be corrected to “Affiliated Hospital of Guangdong Medical University, Zhanjiang, Guangdong, China”.
2. On page 12, “PV6000 (ZSGB, Beijing, China)” should be corrected to “PV6000 (ZSGB‐BIO, Beijing, China)”.

We apologize for these errors. The authors confirmed that all results and conclusions of this article remain unchanged.

